# Cultivation and mating of the truffle *Tuber japonicum* in plantations of ectomycorrhizal *Quercus serrata* seedlings

**DOI:** 10.1128/aem.02362-24

**Published:** 2025-01-10

**Authors:** Noritaka Nakamura, Akihiko Kinoshita, Shota Nakano, Hitomi Furusawa, Keisuke Obase, Muneyoshi Yamaguchi, Kyotaro Noguchi, Yuki Kitade, Takashi Yamanaka

**Affiliations:** 1 Kyushu Research Center, Forestry and Forest Products Research Institute, Forest Research and Management Organization 119808 , Kumamoto, Japan; 2 Forestry and Forest Products Research Institute, Forest Research and Management Organisation 538572 , Tsukuba, Japan; 3 Hokkaido Research Center, Forestry and Forest Products Research Institute, Forest Research and Management Organization 88254 , Sapporo, Japan; 4 Tohoku Research Center, Forestry and Forest Products Research Institute, Forest Research and Management Organization 52770 , Morioka, Japan; Royal Botanic Gardens, Surrey, United Kingdom

**Keywords:** truffle, cultivation, mating, microsatellite

## Abstract

*Tuber japonicum*, a white-colored truffle that is endemic to Japan, is promising for culinary purposes due to its unique aroma. We were able to cultivate *T. japonicum* in plantations of inoculated *Quercus serrata* seedlings for the first time. Ascocarps were found after 43 months at one site and after 61 months at another. We developed simple sequence repeat markers for multilocus genotyping of glebal tissue and ascospores and confirmed that the harvested ascocarps were derived from inocula. All paternal individuals matched the multilocus genotypes of neighboring maternal individuals, indicating frequent hermaphroditism and the absence of externally introduced individuals. Our findings provide important clues to understanding the reproductive biology of *T. japonicum* during the early period after the truffle plantation establishment.

**IMPORTANCE:**

Truffles are highly prized as a delicacy, but only a select few species have been successfully cultivated. In our study, we succeeded for the first time in cultivating *Tuber japonicum*. Two out of four plantations produced ascocarps shortly after planting, with one of them yielding a comparable weight to other cultivated truffle species. This promising productivity suggests that the fungus has potential when cultivated. Our analysis of the ascocarps' maternal and paternal genotypes, using simple sequence repeat markers, revealed hermaphroditic behavior in the fungus at our planting site. Our findings provide crucial insights into the truffle mating events.

## INTRODUCTION

*Tuber* spp. are ectomycorrhizal fungi whose fruiting bodies are highly valued for culinary uses due to their unique aroma (1). Some truffle species, such as *Tuber melanosporum*, *Tuber aestivum*, and *Tuber borchii*, have been cultivated (2). In some of the first approaches established for truffle cultivation, host plant seeds were planted in truffle-growing areas to obtain truffle-colonized seedlings, which were transplanted to develop new plantations (1). Since the 1960s, seedlings have been inoculated with a suspension of ascospores (1). Today, several truffle species are being cultivated worldwide, mainly by spore inoculation (2).

The mycorrhizae of *T. melanosporum*, which supply nutrients to ascocarps (3), are haploid and carry one of two different mating-type genes (4), and ascocarps result from mating between two different individuals (5, 6) harboring opposite mating type genes (heterothallism).The formation of ascocarps through the mating of two distinct individuals was also confirmed in *T. magnatum* (7), *T. aestivum* (8), and *T. borchii* (9) by genetically analyzing the gleba and the asci and ascospores. Also, analyses of mating-type genes in several species including *T. melanosporum* (6), *T. indicum* (10), *T. borchii* (11), *T. aestivum* (12), *T. himalayense*, and *T. longispinosum* (13) further substantiated the heterothallic nature of these fungi. Among the two individuals that constitute the ascocarps, one predominantly contributes to the glebal tissue and is referred to as the maternal individual, which genetically corresponds to the neighboring mycorrhizae (4, 14). Conversely, the paternal individual is solely detectable by the genetic analysis of ascospores (5–9, 15, 16). The individuals that contributed the paternal genetic material usually have not been detected in neighboring mycorrhizae, and their contribution for mating limited for only a year in many cases (8, 15, 16). Thus, although *Tuber* individuals have the potential to play both maternal and paternal roles in different ascocarps, only a limited number of individuals function as hermaphrodites in fields. Based on the differences between maternal and paternal status, it is hypothesized that paternal individuals occupy a different ecological niche than maternal ones. A prominent hypothesis suggests that paternal gametes originate from ascospores (9, 15–17). Additionally, mitospores have been discovered in several species, including *T. japonicum*, and it is hypothesized that they function as spermatia in the mating process (18–21). Nevertheless, to date, no definitive evidence has been acquired to determine the origin of the paternal gametes, and our understanding of the mating system of these fungi remains incomplete.

*Tuber japonicum*, the iconic species of the Japonicum clade in the genus *Tuber*, occurs exclusively in Japan (22), and close relatives, for example, *T. turmericum*, *T. xanthomonosporum*, and *T. flavidosporum* have been discovered in China and Japan (22–25). The mature ascocarps are relatively large (the maximum weight of 101.6 g per ascocarp; Nakamura, pers. obs.), and emit a garlic-like odor produced by volatile compounds such as 3-methyl-2,4-dithiapentane, similar to the white truffle *T. magnatum* (26). We anticipate that *T. japonicum* will be cultivated commercially owing to these advantageous characteristics and high yields in its natural habitats, but to date, this fungus is not being cultivated. Cultivation trials have recently been initiated and the soil conditions of its natural habitats (27), host specificity and mycorrhizal morphology (28), culture conditions (29, 30), and anamorphic morphology (21) have been investigated. As part of these series of studies, in November 2022, we collected ascocarps at two of four sites where ectomycorrhizal seedlings inoculated with *T. japonicum* were planted in October 2017 and April 2019. The aim of this study was twofold: (i) to document the characteristics of sites where ascocarps formed, and (ii) to obtain fundamental information on the mating that occurred at the sites.

## MATERIALS AND METHODS

### Production of *Quercus serrata* seedlings inoculated with *T. japonicum*

Seedlings of *Quercus serrata* Thumb were grown in a sterilized pumice (Kanuma soil) in a temperature-controlled (around 25°C), semi-enclosed room with transparent polycarbonate walls built in a greenhouse to prevent contamination by other mycorrhizal fungi. The room was illuminated by both sunlight and fluorescent lamps. The plants were watered once or twice a week.

The seedlings were inoculated with spores of *T. japonicum* as follows. Ascocarps of *T. japonicum* were collected from Kobe City, Hyogo Prefecture and Inabe City, Mie Prefecture (hereafter Hyogo ascocarps and Mie ascocarps, respectively) for seedling inoculation. The peridium of the ascocarps was excised before immersing the glebal tissue in distilled water in a mortar and crushing it with a pestle. The resulting slurry was filtered through a tea strainer (mesh size approx. 700 µm) and tissue paper (KimWipes S200; NIPPON PAPER CRECIA, Tokyo, Japan) and the spore concentration was adjusted to 1.0 × 10^5^–1.0 × 10^6^/mL by using a hemocytometer (Kayagaki-Irika-Kogyo, Tokyo, Japan). Each pot was inoculated with 1.0 mL of spore suspension. The plants were watered once or twice a week. The formation of mycorrhizae was verified with stereomicroscopy at 240 days after inoculation. Eventually, 16 and 17 seedlings were produced, inoculated with Mie and Hyogo inoculum, respectively.

These mycorrhizal seedlings (hereafter “mother seedlings”) were propagated utilizing the “mother plant” technique (31). Mother seedlings were transplanted into larger containers filled with sterilized pumice (Hyuga soil) and nourished with 50 mL of Universol Blue fertilizer (666.6 mg/L) per container. After growing for 90 days, each seedling was positioned in the center of a plastic Wagner pot (16 cm diameter, 25 cm height). Eight to 10 sterilized *Q. serrata* seedlings, hereafter “recipient seedlings,” were placed around the mother seedlings to ensure root contact. The plants were watered once or twice a week. After 1 month, mycorrhizal formation on the recipient seedlings was confirmed by stereomicroscope, and they were relocated to new plastic pots containing pumice (Hyuga soil) and treated with 50 mL of Universol Blue fertilizer (666.6 mg/L) per pot. Eventually, more than 40 inoculated seedlings were produced for each inoculum.

### Establishment of truffle plantations

**Fig 1:**
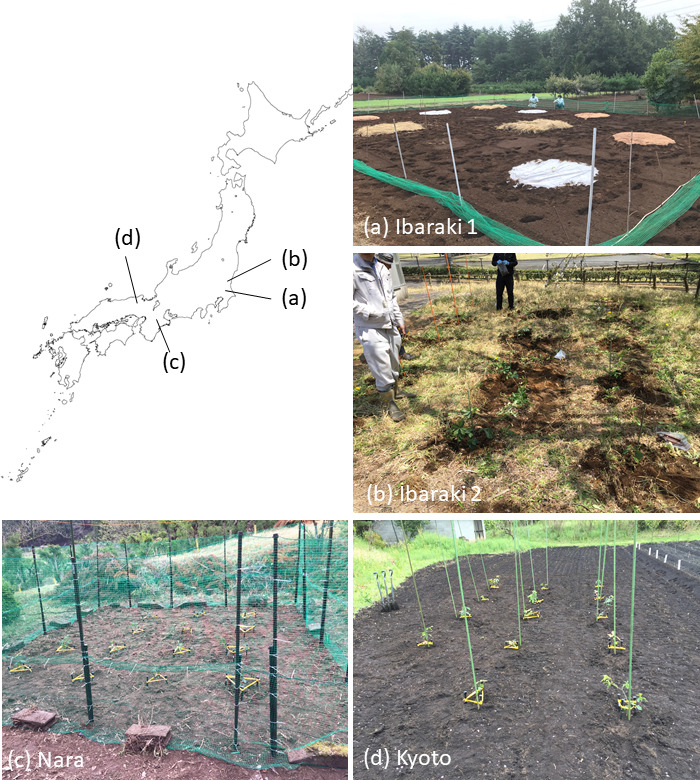
Four locations where *Tuber japonicum*-inoculated seedlings were planted. Photographs of the plantations in (a) Ibaraki 1, (b) Ibaraki 2, (c) Nara, and (d) Kyoto are also presented. Blank map from https://www.freemap.jp/.

Truffle plantations were established in four distinct locations: two within Ibaraki Prefecture in eastern Japan (Ibaraki 1 and 2 sites) and one each in Nara and Kyoto Prefectures in western Japan (Nara and Kyoto sites) (Fig. 1; Table 1). To protect these sites, their precise locations have been omitted from the present paper. To minimize contamination by other ectomycorrhizal fungi, all four sites were selected based on the absence of any records of ectomycorrhizal host plants in the preceding vegetation. All sites were located in the humid temperate climatic zone (Cfa) of the Köppen climate classification.

**TABLE 1:** Information on the four sites where seedlings inoculated with *Tuber japonicum* were planted^*a*^

Site name	Location	Site size	Annual average soil temperature	Soil pH	FAO soil classification	Planting date	No. of plants	Distance among plants (m)	No. of plants on Nov. 2022	Average height of plants	No. of ascocarps harvested in Nov. 2022
Ibaraki 1	Tsukuba city, Ibaraki Pref.	12 m × 16 m	16.1	5.7	Cambisol	3 Oct 2017	12	4	10	276	8
Ibaraki 2	Naka city, Ibaraki Pref.	6 m × 3 m	N/A^*b*^	6	Regosol	22 Apr 2019	14	1	11	70	0
Kyoto	Fukuchiyama city, Kyoto Pref.	9 m × 5 m	16	5.7	Andosol	26 Apr 2019	14	1.5	14	253	14
Nara	Uda city, Nara Pref.	5 m × 5 m	13.4	5.5	Arenosol	25 Apr 2019	14	1	14	67	0

^*a*^ Average height of plants was measured in November 2022 (Ibaraki 1, 2, and Kyoto) and June 2023 (Nara).

^*b*^ N/A indicates data not available.

In Ibaraki 1 site, 12 *Q. serrata* seedlings (270 days post-sowing; inoculated with Hyogo or Mie ascospores, six seedlings each) were planted on October 3, 2017, within the plot at 4 m intervals. One of four mulching treatments was applied to each seedling: mulching with non-woven fabric sheets, rice straw, wood chips, or no mulch (three seedlings each). Mulch was dispersed within a 1 m radius surrounding the seedlings. Since two of the three seedlings mulched with wood chips died by October 2018, the wood chips were removed from the one surviving seedling in May 2019. No significant differences in plant growth were observed in the other treatments; therefore, the mulch treatment will not be discussed further. No fertilizer was applied after planting. Root fragments were randomly sampled on October 16, 2018 and October 24, 2019, to investigate the formation of mycorrhizae.

Mycorrhizae were observed under a stereomicroscope, and those similar to *T. japonicum* mycorrhizae were selected based on mantle color and the presence of cystidia (28). The selected mycorrhizae were identified based on the ITS region sequence of the rDNA. Soil on the surface of the excised mycorrhizae was removed under a stereomicroscope, and the mycorrhizal DNA was extracted using the Kaneka Easy DNA Extraction Kit version 2 (Kaneka Corporation, Tokyo and Osaka, Japan). However, all liquid volumes used were one-tenth of the manufacturer’s protocol. DNA extracts were diluted 1/10 and used for PCR. The PCR primer pair ITS1F (50-CTTGGTCATTTAGAGGAAGTAA-30) (32) and ITS4 (50-TCCTCCGCTTATTGATATGC-30) (33) was used to carry out PCR amplification with Tks Gflex DNA Polymerase (Takara Bio, Kusatsu, Japan) according to the manufacturer’s protocol. The PCR conditions were as follows: initial denaturation at 95°C for 5 min; followed by 35 cycles of denaturation at 95°C for 30 s, annealing at 55°C for 30 s, and extension at 68°C for 40 s; with a final extension at 68°C for 5 min. The PCR products were purified with Exonuclease I and Antarctic phosphatase (New England Biolabs, Ipswich, MA, USA) (34). Cycle sequencing was performed with a BigDye Terminator (v3.1) Cycle Sequencing Kit (Thermo Fisher Scientific), according to the manufacturer’s protocol. DNA sequences were determined using an ABI PRISM 3130xl Genetic Analyzer (Thermo Fisher Scientific).

In the other three sites (Ibaraki 2, Nara, and Kyoto sites), 14 *Q. serrata* seedlings were planted on April 22 (Ibaraki 2), April 25 (Nara), and April 26 (Kyoto), 2019. The seedlings were prepared by using the mother plant technique, using seedlings inoculated with either Hyogo (five per site) or Mie (nine per site) ascocarps as mother plants. No mulching treatment was applied.

We visited the sites to search for ascocarps in 2022, on November 21 (Nara), November 22 (Kyoto), November 25 and November 28 (Ibaraki 1), and November 29 (Ibaraki 2). The precise location of each ascocarp within the respective sites was recorded. The ascocarps were placed in separate plastic bags and transported to the laboratory where they were weighed after removing any attached soil.

### DNA extraction from harvested ascocarps

In previous molecular analyses, most glebal tissue samples contained a single genotype (i.e., maternal individuals), whereas the genotypes of both parents were detected in the ascospores (5–7). Thus, we extracted DNA from both glebal tissues and ascospores. Glebal tissue samples (3 mm cubes) were excised from the interior of ascocarps and crushed with a pestle. Subsequent DNA extraction was performed using the CTAB method (35).

For DNA extraction from ascospores, mature ascocarps stored at 4°C for at least 1 month were utilized. Glebal tissues were sectioned into approximately 1 cm cubes, thoroughly ground in a mortar and pestle, and filtered through tissue paper (KimWipes S200) to prepare a spore suspension which was then transferred into microtubes at 2 mL per tube. The spores were collected by centrifugation and were resuspended in 150 µL of 2× CTAB buffer. Zirconia beads with a diameter of 5 mm (Toray Industries, Tokyo, Japan) were added to the microtubes and fungal cells, including ascocarps, were disrupted using a Multi-beads Shocker (Yasui Kikai Corporation, Osaka, Japan) at 3,000 rpm for 30 s. Subsequent DNA extraction was performed using the CTAB method.

DNA was extracted from two strains (S74-2 and KobeB) originating from the same natural truffle grounds along with the inocula. These strains were cultivated in 20 mL of liquid Ma/2 medium, comprising 10 g glucose, 10 g malt extract, 1 g peptone, and 1 L deionized water (36), at 25°C over a period of 2 weeks. Subsequently, the cultures were rinsed with sterilized water, and excess moisture was gently removed using a Kim wipe. The DNA extraction from the cultures was performed using the CTAB method, as previously outlined.

### Design of primers for mating-type genes

The mating type genes in *T. japonicum* were identified using the following method. Briefly, one of the two mating-type gene regions was identified from the genomic data of a selected strain (strain S64-2) from the Forestry and Forest Products Research Institute (FFPRI)’s collection of *T. japonicum* strains, based on its similarity to the mating-type gene sequences of *T. indicum*, *T. himalayense*, and *T. longispinosum*. Subsequently, PCR primers were designed to amplify a segment of the mating-type gene. Following this, strains lacking this gene were selected from the FFPRI collection of strains using the aforementioned PCR primers, and genome sequencing was performed on one of these strains (strain S20-1). From this genomic data, the opposite mating-type gene region was identified. Finally, multiplex-PCR primer pairs were redesigned to specifically amplify the portions of the genes *MAT1-1-1* and *MAT1-2-1*.

*T. japonicum* strain S64-2 was cultivated in liquid-modified Melin-Norkrans medium (MMN; 37) at 25°C for 2 weeks. Genomic DNA was extracted using the CTAB method and genome sequencing and assembly was outsourced to Macrogen (Macrogen Inc., Seoul, Republic of Korea). Library preparation was performed using a True Seq Nano kit (Illumina, San Diego, CA, USA) to construct a library and subsequently analyzed using the NovaSeq 6000 platform with 151 bp paired-end reads (Illumina). *De novo* assembly was performed by using SOAPdenovo2 (38). The completeness of the assembled genome was evaluated using BUSCO v5 (39) with the Fungi ortholog set in the gVolante web interface (40).

The part of the *T. japonicum MAT1-2-1* gene region was identified from the strain S64-2 by performing local BLAST searches of the assembled sequence data using the tblastn program, with the GenBank amino acid sequences of *MAT1-2-1* from *T. indicum*, *T. himalayense*, and *T. longispinosum* (BBB16413, BBB16410, and BBB16430) as queries. A primer pair (TjMAT2_f1: 5′-GTCACGGATGATGCTAAGGCTAGC-3′; TjMAT2_R2: 5′-TTCCAGTCAACTGGAAGCATACG-3′) was designed to amplify a specific region only to selectively identify strains with distinct mating-type genes from the strain collection. The PCR conditions were as follows: initial denaturation at 95°C for 3 min; followed by 30 cycles of denaturation at 95°C for 20 s, annealing at 55°C for 20 s, and extension at 68°C for 40 s; with a final extension at 68°C for 5 min. Within the *T. japonicum* strain collection at the FFPRI, strain S20-1 was chosen for additional genome sequencing due to the lack of PCR amplification with the primers, suggesting it harbors the opposite mating type gene (*MAT1-1-1*). We used the abovementioned method to perform genome sequencing of the S20-1 strain and search for the mating-type gene. A local BLAST using the tblastn program was conducted utilizing the amino acid sequence of the *MAT1-1-1* gene of *T. borchii* (PUU82705) as a query to identify a potential *MAT1-1-1* gene with high affinity, using BioEdit Ver. 7.2.6 (41). We confirmed that these regions are idiomorphs with well-conserved flanking regions using sequencing alignment among these two strains.

### Determination of maternal individuals’ mating types

**Fig 2:**
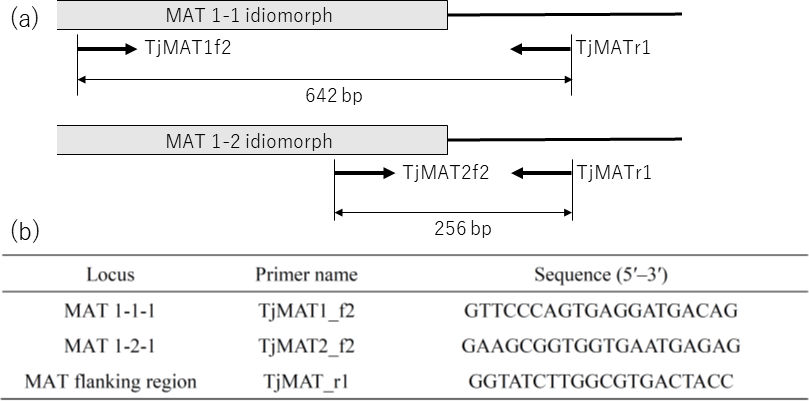
Primer pairs utilized in this study to determine the mating types of ascocarps. (**a**) The primers TjMAT1f2 and TjMAT2f2 were designed to anneal to MAT1-1 and MAT1-2 idiomorphs, respectively, while TjMATr1 was designed to anneal to the MAT flanking region. (**b**) List of targeted loci and sequences of the primers.

The mating type of maternal individuals was ascertained via multiplex PCR, utilizing DNA extracted from the glebal tissue as the template and the abovementioned primers targeting *T. japonicum* mating-type genes. Primer triplets designed for multiplex PCR were developed to determine mating types in a single PCR reaction (Fig. 2). To validate the efficacy of this primer set, PCR was conducted using DNA from the cultured strains listed in Table S1. The strains were cultured in liquid Ma medium at 25°C for 2 weeks and the genomic DNA was extracted from these strains using CTAB method as described above. Exclusively the primer set depicted in Fig. 2 were employed to identify the maternal mating types of the ascocarps. Multiplex PCR was conducted using Multiplex PCR Assay Kit Ver.2 (Takara Bio) following the manufacturer’s protocol. The PCR conditions were as follows: initial denaturation at 95°C for 3 min; followed by 35 cycles of denaturation at 95°C for 10 s, annealing at 55°C for 10 s, and extension at 72°C for 20 s; with a final extension at 72°C for 5 min. PCR products were subjected to gel electrophoresis using a Mupid-exU system (Mupid, Tokyo, Japan) and the resulting electropherogram was visualized using a GelDoc Go Imaging System (Bio-Rad, Hercules, CA, USA).

### Development of simple sequence repeat primers and analysis of SSR loci

Based on the 4,362 assembled contigs of strain S64-2, PCR primer pairs were developed to amplify the simple sequence repeat (SSR) regions determined by using the Krait software (42). Of the 29,734 loci detected, 82 primer pairs were arbitrarily selected based on the PCR annealing temperature (>60°C) and the number of motif repetitions (>3). For each primer pair, PCR amplification was verified for 23 ascocarp specimens collected in Japan (Table S2). PCR reactions were conducted using Takara Ex Taq (Takara Bio) in accordance with the manufacturer’s protocol. PCR products were analyzed via 1.0% agarose gel electrophoresis and stained with SYBR Green I Nucleic Acid Gel Stain (Takara Bio). For primer pairs with positive PCR amplification in most specimens, one of four fluorescent dyes (FAM, NED, PET, and VIC) was attached to the 5′ end of the forward primers (ThermoFisher Scientific, Waltham, MA, USA), and a pigtail sequence (5′-GTTTCTT-3′; 43) was attached to the 5′ end of the reverse primers to facilitate adenylation and ensure precise genotyping (Table S3). SSR polymorphisms were analyzed with multiplex PCR using the Takara Multiplex PCR Assay Kit (Takara Bio). The following PCR protocol was used: initial denaturation at 95°C for 1 min; followed by 40 cycles of denaturation at 95°C for 30 s, annealing at 60°C for 90 s, and extension at 72°C for 30 s; with a final extension at 60°C for 30 min. PCR products were diluted 100-fold and 0.5 µL of the diluted PCR products was dissolved in 10 µL of Hi-Di Formamide (ThermoFisher Scientific) with 0.5 µL of GeneScan 600 LIZ Dye Size Standard v2.0 (ThermoFisher Scientific). The length of the amplified PCR fragments was determined using a 3500 Genetic Analyzer (ThermoFisher Scientific). The SSR data were analyzed in the software R (44) with the “poppr” package (45). Statistical data for each locus were calculated using the Locus_table function. A genotype accumulation curve for 23 samples (Table S3) was generated through 999 permutations employing the genotype_curve function.

## RESULTS

### Host planting and ascocarp occurrence

**Fig 3:**
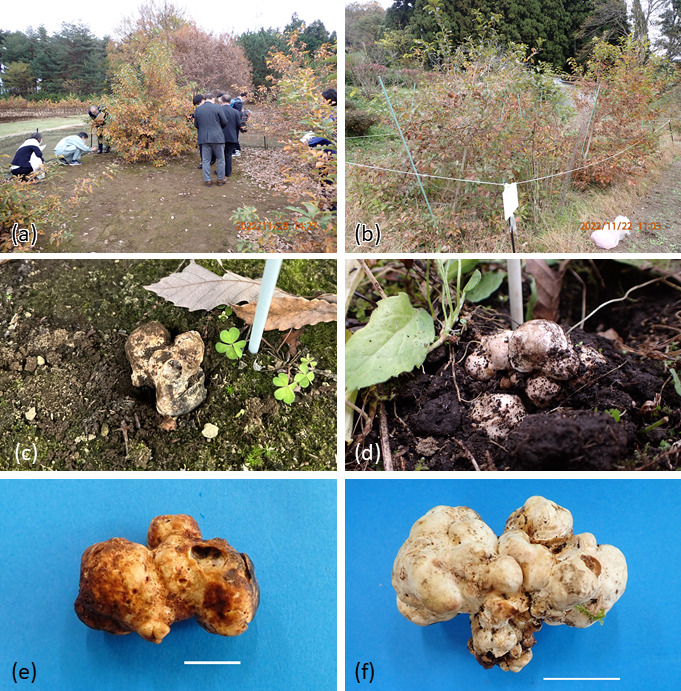
Productive truffle plantations and fruiting bodies photographed in November 2022. (**a**) Ibaraki 1 site, (**b**) Kyoto site, ascocarps emerging from the soil surface at (**c**) Ibaraki 1 and (**d**) Kyoto sites, and harvested ascocarps collected from (**e**) Ibaraki 1 and (**f**) Kyoto sites. Bars in (**e**) indicate 1.5 cm and (**f**) 3 cm.

**Fig 4:**
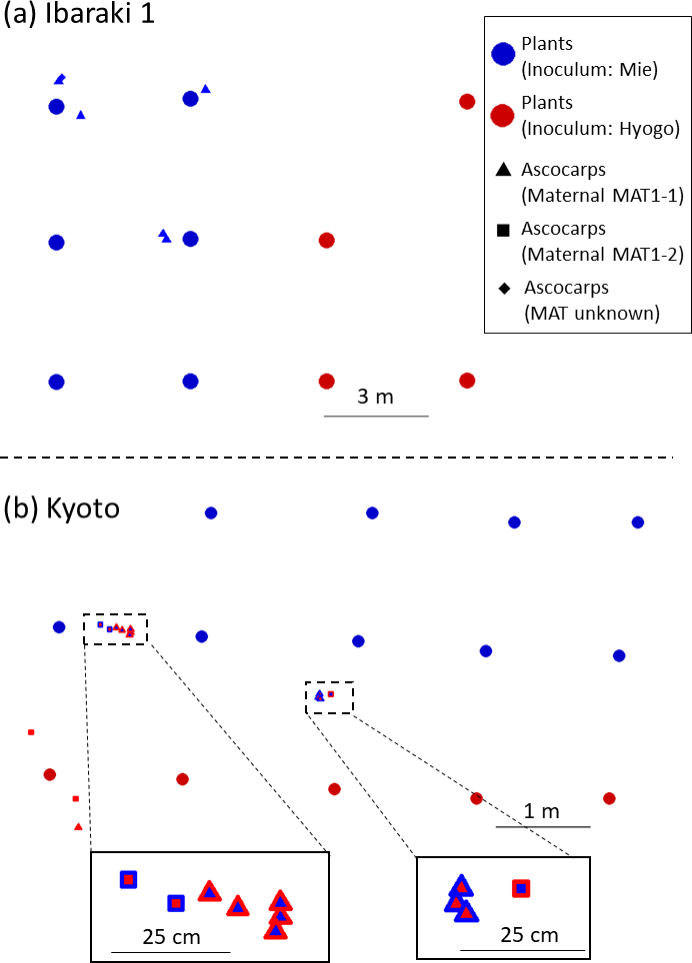
Location of plants and ascocarps at (**a**) Ibaraki 1 and (**b**) Kyoto sites. Enlarged views where ascocarps aggregated are shown in (**b**). Plant positions are indicated by filled circles. Harvested ascocarps are indicated by triangles (maternal mating type: MAT1-1) or squares (maternal mating type: MAT1-2). Blue indicates simple sequence repeat–multilocus genotype (SSR-MLG) 1 (from Mie) and red indicates SSR-MLG 2 (from Hyogo). For each symbol, the outer color represents the maternal SSR-MLG and the inner color represents the paternal SSR-MLG. Since two plants were dead, only the spatial positions of the 10 survived plants are depicted in (**a**).

Formation of mycorrhizae was observed in mother seedlings within 240 days after inoculation and in recipient seedlings at 30 days. Planting date, average height of plants in 2022, and site status are shown in Table 1. Root samples randomly collected from the Ibaraki 1 site on October 16, 2018 and October 24, 2019 consistently contained *T. japonicum* mycorrhizae, indicating the persistence of the fungus. Ascocarps observed on the soil surface were collected on November 22, 2022, in Kyoto site and on November 25 and 28, 2022, in Ibaraki 1 site (Fig. 3); no ascocarps were found at either Nara and Ibaraki 2. In Kyoto, 14 ascocarps were harvested, weighing a total of 266.5 g. In Ibaraki 1 site, eight ascocarps were harvested, weighing a total of 38.6 g. The locations of the ascocarps at each site are depicted in Fig. 4. At Ibaraki 1 site, ascocarps occurred solely around plants inoculated with Mie inoculum, whereas at Kyoto, ascocarps occurred around plants inoculated with both types of inoculum, but their distribution was not spatially uniform (Fig. 4).

### Development of mating type-specific primers and SSR markers

The assembled genomes had total lengths of 117 and 130 Mbp, with *N*_50_ sequence lengths of 73,123 and 89,536 for the strains S20-1 and S64-2, respectively. Among the 758 core genes of the Fungi ortholog set, 732 (96.57%) and 735 (96.97%) complete genes were detected in the strains S20-1 and S64-2, respectively. Utilizing primers designed from the DNA sequences of the mating-type genes, one of the mating types was detected from each strain (Fig. S2a), thereby confirming the primers’ efficacy.

Of the 82 SSR primer pairs tested, 11 amplified polymorphic target regions (Table S3). DNA from 23 fruiting body samples collected from various regions in Japan was analyzed (Table S2). The number of alleles at each locus varied from 3 (ssr-152) to 10 (ssr-18881), with an average of 5.73. The probability that two arbitrarily chosen samples would exhibit different alleles, 1-D, averaged 0.58 and with an average evenness of 0.63 (Table S3). We constructed a genotype accumulation curve, which indicated that data from six arbitrarily chosen loci was sufficient to identify >90% of the 22 observed multilocus genotypes (MLGs); data from 11 loci would therefore be sufficient to identify the individuals in our study (Fig. S1).

### SSR genotypes and mating types of the harvested ascocarps

Regrettably, during cultivating the plants, the inocula themselves were lost, rendering their direct analysis impossible. Consequently, we analyzed two strains (S74-2 and KobeB), which derived the same natural truffle ground as the inocula. Our assumption was that these strains originated from the identical genets as the inocula. Strain S74-2 from Mie corresponded to SSR-MLG 1, while KobeB from Hyogo represented SSR-MLG 2 (see Table 2)

**TABLE 2:** Maternal and paternal simple sequence repeat (SSR)–multilocus genotypes (MLGs) and maternal mating types of harvested and inoculum ascocarps^*a*^

Ascocarp number	Site	Maternal (M) or paternal (P)	PCR product length (bp) for each SSR locus												SSR-MLG	Mating type
			ssr-18861	ssr-15233	ssr-152	ssr-4908	ssr-3313	ssr-20732	ssr-9116	ssr-18177	ssr-18881	ssr-12988	ssr-20435	ssr-21085		
A-1	Kyoto	M	142	234	174	235	152	221	151	143	204	221	120	161	2	MAT1-2
		P	142	234	174	235	152	221	151	143	204	221	120	161	2	
A-2	Kyoto	M	142	234	174	235	152	221	151	143	204	221	120	161	2	MAT1-1
		P	142	234	174	235	152	221	151	143	204	221	120	161	2	
B-1	Kyoto	M	142	234	174	235	152	221	151	143	204	221	120	161	2	MAT1-2
		P	142	234	174	235	152	221	151	143	204	221	120	161	2	
C-1	Kyoto	M	146	244	171	245	160	218	155	143	184	224	120	161	1	MAT1-2
		P	142	234	174	235	152	221	151	143	204	221	120	161	2	
D-1	Kyoto	M	146	244	171	245	160	218	155	143	184	224	120	161	1	MAT1-2
		P	142	234	174	235	152	221	151	143	204	221	120	161	2	
D-2	Kyoto	M	142	234	174	235	152	221	151	143	204	221	120	161	2	MAT1-1
		P	146	244	171	245	160	218	155	143	184	224	120	161	1	
D-3	Kyoto	M	142	234	174	235	152	221	151	143	204	221	120	161	2	MAT1-1
		P	146	244	171	245	160	218	155	143	184	224	120	161	1	
E-1	Kyoto	M	142	234	174	235	152	221	151	143	204	221	120	161	2	MAT1-1
		P	146	244	171	245	160	218	155	143	184	224	120	161	1	
E-2	Kyoto	M	142	234	174	235	152	221	151	143	204	221	120	161	2	MAT1-1
		P	146	244	171	245	160	218	155	143	184	224	120	161	1	
E-3	Kyoto	M	142	234	174	235	152	221	151	143	204	221	120	161	2	MAT1-1
		P	146	244	171	245	160	218	155	143	184	224	120	161	1	
F-1	Kyoto	M	146	244	171	245	160	218	155	143	184	224	120	161	1	MAT1-1
		P	142	234	174	235	152	221	151	143	204	221	120	161	2	
F-2	Kyoto	M	146	244	171	245	160	218	155	143	184	224	120	161	1	MAT1-1
		P	142	234	174	235	152	221	151	143	204	221	120	161	2	
F-3	Kyoto	M	146	244	171	245	160	218	155	143	184	224	120	161	1	MAT1-1
		P	142	234	174	235	152	221	151	143	204	221	120	161	2	
F-4	Kyoto	M	142	234	174	235	152	221	151	143	204	221	120	161	2	MAT1-2
		P	146	244	171	245	160	218	155	143	184	224	120	161	1	
T-A	Ibaraki 1	M	146	244	171	245	160	218	155	143	184	224	120	161	1	MAT1-1
		P	146	244	171	245	160	218	155	143	184	224	120	161	1	
T-B	Ibaraki 1	M	146	244	171	245	160	218	155	143	184	224	120	161	1	MAT1-1
		P	146	244	171	245	160	218	155	143	184	224	120	161	1	
T-C	Ibaraki 1	M	146	244	171	245	160	218	155	143	184	224	120	161	1	N/A
		P	146	244	171	245	160	218	155	143	184	224	120	161	1	
T-D	Ibaraki 1	M	146	244	171	245	160	218	155	143	184	224	120	161	1	MAT1-1
		P	146	244	171	245	160	218	155	143	184	224	120	161	1	
T-E	Ibaraki 1	M	146	244	171	245	160	218	155	143	184	224	120	161	1	MAT1-1
		P	146	244	171	245	160	218	155	143	184	224	120	161	1	
T-F	Ibaraki 1	M	146	244	171	245	160	218	155	143	184	224	120	161	1	MAT1-1
		P	146	244	171	245	160	218	155	143	184	224	120	161	1	
T-G	Ibaraki 1	M	146	244	171	245	160	218	155	143	184	224	120	161	1	MAT1-1
		P	146	244	171	245	160	218	155	143	184	224	120	161	1	
T-H	Ibaraki 1	M	146	244	171	245	160	218	155	143	184	224	120	161	1	MAT1-1
		P	146	244	171	245	160	218	155	143	184	224	120	161	1	
S74-2	Inabe, Mie	M	146	244	171	245	160	218	155	143	184	224	120	161	1	N/A^*b*^
KobeB	Kobe, Hyogo	M	142	234	174	235	152	221	151	143	204	221	120	161	2	N/A^*b*^

^*a*^ Maternal and paternal genotypes were determined based on the genotypes of solely glebal tissue, and a combination of genotypes of glebal tissue and ascospores, respectively.

^*b*^ N/A indicates data not available.

The mating types of maternal individuals and the spatial distribution of the SSR-MLGs for maternal and paternal individuals are illustrated in Fig. 4. Overall, we identified two SSR-MLG types, SSR-MLG 1 and 2. For each maternal SSR-MLG, we observed different mating types, indicating that at least four genetically distinct individuals were present at the sites. There were differences at nine loci between SSR-MLG 1 and 2, and no recombinants were detected. The DNA sequencing analysis confirmed that all multiplex PCRs for mating type analysis, except for that of T-C, successfully amplified either of the mating type gene regions. Presumably, due to poor DNA quality, the maternal mating type of T-C could not be determined.

At Ibaraki 1 site, only SSR-MLG 1 was detected in both maternal and paternal genotypes, indicating that both parents of the ascocarps were SSR-MLG 1. The maternal individuals of all ascocarps, except for T-C, exhibited a mating type of MAT1-1.

At Kyoto site, the maternal SSR-MLGs were identified as either SSR-MLG 1 (five ascocarps) or SSR-MLG 2 (nine ascocarps; Table 2). Also, the paternal SSR-MLGs were identified as either SSR-MLG 1 (six ascocarps) or SSR-MLG 2 (eight ascocarps). In 11 of the ascocarps at Kyoto site, both SSR-MLGs were detected as either of the parents, resulting from mating events between maternal SSR-MLG 1 and paternal SSR-MLG 2 or vice versa; in the remaining three ascocarps, both parents were SSR-MLG 2. Mating type analysis revealed that the maternal partners of nine ascocarps were MAT1-1, while those of five ascocarps were MAT1-2 (Table 2; Fig. S2b).

## DISCUSSION

### First successful cultivation of the truffle *T. japonicum* in Japan

This is the first report of ascocarp development from field-grown truffle-inoculated seedlings in Japan and the first successful cultivation of *T. japonicum*. Typically, the first truffles are harvested at 5–10 years post-planting (1); however, ascocarp development was achieved at one site at only 3.5 years post-planting. As far as we know, this is close to the shortest time to truffle harvest (3 years; (1)). In Kyoto, 266.4 g of ascocarps was harvested, which corresponds to a presumed production of 59.2 kg/ha (site size = 45 m^2^); however, the Ibaraki 1 site produced only 38.6 g of ascocarps. The value at Kyoto site is comparable to the production at well-managed *T. melanosporum* plantations (46); however, it is extrapolated from a small experimental site and therefore this comparison must be regarded with caution. These results suggest that this fungus is comparable to that of *T. melanosporum* in terms of ascocarps productivity. Production of *T. japonicum* ascocarps at Ibaraki 1 and Kyoto sites—both of which were well exposed to the sun (data not shown) and had apparently well-developed seedlings (average plant height of 276 and 253 cm) compared to the other two sites (67 and 70 cm; Table 1)—implies that an adequate supply of photosynthates from the host plants might be one of the factors determining productivity.

Among the four plantations developed in this study, spore-inoculated seedlings were planted in only one (Ibaraki 1) site, while the remaining sites employed the mother plant technique for inoculation. Spore inoculation is the most commonly used method for producing infected plants (1, 2, 47). The mother plant technique is an effective method as it requires a short period of time (1 month) for infection as reported in this study; however, there is a risk of using contaminated mother plants, which could spread contamination to uninfected plants (47, 48). In this study, we successfully harvested ascocarps from plants produced using the mother plant technique, potentially due to minimizing contamination by utilizing a closed room in the greenhouse. Although there have probably been previous instances of truffle production using this inoculation method, no examples of truffle harvesting appear to have been published, despite its similarity to Talon’s technique, the earliest method for cultivating *T. melanosporum* (1). Therefore, to the best of the author’s knowledge, this is the first report of truffle production using seedlings infected with the mother plant technique.

### Newly developed mating types and SSR markers

Evaluation of the genome assemblies from the two *T. japonicum* strains using BUSCO revealed that the majority (>96.5%) of the core genes in the Fungi data set were present in both assemblies, indicating that these data are adequate for the development of genetic markers. The *T. japonicum* mating-type genes were identified for the first time in this study. Pure cultures of *T. melanosporum* (6), *T. indicum* (10), *T. borchii* (11), *T. aestivum* (12), *T. himalayense*, and *T. longispinosum* (13) possess one of the two mating-type genes and are classified as heterothallic. In this study, the two strains utilized for genome sequencing, along with all 11 additional strains, exhibited one of the two mating-type genes (Fig. S2a), corroborating that this fungus is heterothallic, akin to those *Tuber* species. The SSR markers differentiated 22 SSR-MLGs in samples from different locations in Japan (Table S2), indicating that 11 markers were sufficient to differentiate MLGs among these samples. The fungal strains obtained from ascocarps collected in the natural truffle grounds of Hyogo and Mie Prefectures exhibited two distinct SSR-MLGs 1 and 2, differing at eight loci.

### Maternal and paternal individuals involved in ascocarp formation

The SSR analysis revealed that the maternal individuals of all ascocarps corresponded to one of the two strains: S74-2 and KobeB (Table 2). Although we could not directly analyze the inocula themselves due to their loss, the perfect correspondence between harvested ascocarps and these strains, along with the consistent occurrence of SSR-MLG 1 in Ibaraki 1 and Kyoto sites, strongly supports that the ascocarps originated from the inocula. In Kyoto site, both SSR-MLG 1 and SSR-MLG 2 were detected as maternal individuals, and in some ascocarps that formed near plants inoculated with Mie ascocarps (presumably SSR-MLG 1) the maternal tissue was SSR-MLG 2 (Fig. 4), indicating that the maternal individual expanded over 170 cm from its original location.

We found both mating types in each maternal SSR-MLG, indicating that at least two individuals were present for each SSR-MLG. The original inocula were unavailable for SSR analysis, but this result suggests that the inocula were highly homozygous, likely due to repeated sibling mating. The occurrence of homozygous ascocarps in both natural truffle grounds and in plantations was repeatedly observed in *T. magnatum* (7) and *T. melanosporum* (4, 15, 16). Thus, it is not necessarily unique that the inocula derived from the natural truffle grounds we used in this study were homozygous at all SSR loci. Indeed, all ascocarps at Ibaraki 1 and three at Kyoto site, respectively, resulted from mating between individuals of the same SSR-MLG (Table 2; Fig. 4), suggesting that siblings from the same homozygous inocula were involved in the mating process.

### Mating of *T. japonicum* at the sites

Mycorrhizae of *Tuber* spp. are formed by haploid mycelia (14, 49), and both mating types can potentially contribute to the formation of ascocarps as either the maternal (fruiting body forming) or paternal (genetically contributing to ascospores) partner (hermaphroditism). Although analyses of field-collected ascocarps revealed that, in many cases, individual fungi are detected either as paternal or maternal ones, not as both (8, 9, 15, 16) despite their potential hermaphroditism. Furthermore, the annual contribution of paternal partners to ascocarp formation compared to the prolonged contribution by maternal ones over several years suggests distinct niches for paternal and maternal individuals (8, 15, 16). However, we have verified that all the paternal partners at Kyoto site are the same genotype as the maternal partners of adjacent ascocarps, with no intrusion of wild individuals or recombinants among maternal individuals (Table 2). Thus, our results suggest that hermaphroditism was common in Kyoto site. On the other hand, all identified maternal partners represented the same mating type and SSR-MLG in Ibaraki 1 site, suggesting the prevalence of MAT1-1 as maternal partners of the ascocarps. The result in Ibaraki 1 aligns with the previous study of spatial segregation among ascocarps harboring distinct maternal mating types (14). The observed differences between the two sites may be attributed to the narrower distance among plants at the Kyoto site compared to the Ibaraki 1 site (1.5 m vs 4 m; Table 1). It is known that the proportion of mating types of ectomycorrhizae within a root system gradually becomes uneven in *T. melanosporum* (4, 50). Although we did not investigate the ectomycorrhizal mating types, it is possible that the narrower distance between plants in Kyoto site facilitated the mutual migration of the fungus among adjacent plants, thereby increasing the probability of coexistence of different mating types.

In Kyoto site, neighboring individuals with different mating types produced ascocarps at adjacent locations utilizing each other as mating partners (Fig. 4b, enlarged figures). This suggests that the vegetative hyphae or mitospores derived from them participated in mating as maternal and paternal partners. Since the plants at the site were inoculated using the mother-plant technique, the number of ascospores would have been limited and possibly contributed less to mating there. It has been repeatedly hypothesized, but not yet experimentally confirmed, that mitospores function as male gametes in the mating of *Tuber* spp (18–21). Further research is required to determine if mycelium-to-mycelium or mycelium-to-mitospore mating can occur in this fungus using mycelium-inoculated plants.

We observed the first season, or at least the initial phase of productive seasons, of truffle production, as the ascocarps emerged in Kyoto site at 3.5 years and in Ibaraki 1 site at 5 years after planting, with no harvest the preceding year (data not shown). Only ascocarps that could be seen on the soil surface were collected. It is highly probable that some ascocarps remained unharvested, as in *T. melanosporum* (51). It is also conceivable that ascospores were dispersed, as some of the ascocarps displayed feeding marks that may have been caused by insects or other small animals (Fig. 3). If the ascospores or the mycelia germinated from them also participate in mating in this fungus, it is probable that recombinants among introduced individuals (i.e., SSR-MLG 1 and SSR-MLG 2) could be detected as paternal mating partners in subsequent seasons, as observed in reference (9). Transition of the genotypes of the gametes (particularly the paternal ones) would be continuously monitored on our sites.

In this study, we reported for the first time the development of fruiting bodies from seedlings inoculated with *T. japonicum* at two of the four newly developed plantations 41 and 63 months after planting. SSR markers of *T. japonicum* were developed for genetic analysis of the individuals forming ascocarps. Genotyping of glebal tissue revealed that the inoculum contributed to the development of the ascocarps. Analysis of paternal individuals and mating types confirmed that all individuals functioned as hermaphrodites at least at Kyoto site. These results contribute to the understanding of the reproductive biology of *T. japonicum*, particularly during the initial phase of ascocarp production in a newly established plantation.

## Data Availability

The mating-type gene and flanking region sequences identified from the genome have been deposited in the DDBJ database. The accession numbers are LC841963 (*MAT1-1-1*) and LC841964 (*MAT1-2-1*).

## SUPPLEMENTAL MATERIAL

Supplemental material aem.02362-24-s0001.ppt Tables S1 to S3; Fig. S1 and S2.

ASM does not own the copyrights to Supplemental Material that may be linked to, or accessed through, an article. The authors have granted ASM a non-exclusive, world-wide license to publish the Supplemental Material files. Please contact the corresponding author directly for reuse.
